# Do autistic individuals show atypical performance in probabilistic learning? A comparison of cue-number, predictive strength, and prediction error

**DOI:** 10.1186/s13229-025-00651-7

**Published:** 2025-03-04

**Authors:** Jia Hoong Ong, Lei Zhang, Fang Liu

**Affiliations:** 1 Department of Psychology, School of Social Sciences, Nottingham Trent University, Nottingham, UK; 2 Centre for Human Brain Health, School of Psychology, University of Birmingham, Birmingham, UK; 3 Institute for Mental Health, School of Psychology, University of Birmingham, Birmingham, UK; 4 Centre for Developmental Science, School of Psychology, University of Birmingham, Birmingham, UK; 5 School of Psychology and Clinical Language Sciences, University of Reading, Reading, UK

**Keywords:** Probabilistic learning, Associative learning, Prediction errors, Bayesian, Predictive coding, Reinforcement learning

## Abstract

**Background:**

According to recent models of autism, autistic individuals may find learning probabilistic cue-outcome associations more challenging than deterministic learning, though empirical evidence for this is mixed. Here we examined the mechanism of probabilistic learning more closely by comparing autistic and non-autistic adults on inferring a target cue from multiple cues or integrating multiple target cues and learning from associations with various predictive strengths.

**Methods:**

52 autistic and 52 non-autistic participants completed three tasks: (i) single-cue probabilistic learning, in which they had to infer a single target cue from multiple cues to learn cue-outcome associations; (ii) multi-cue probabilistic learning, in which they had to learn associations of various predictive strengths via integration of multiple cues; and (iii) reinforcement learning, which required learning the contingencies of two stimuli with a probabilistic reinforcement schedule. Accuracy on the two probabilistic learning tasks was modelled separately using a binomial mixed effects model whereas computational modelling was performed on the reinforcement learning data to obtain a model parameter on prediction error integration (i.e., learning rate).

**Results:**

No group differences were found in the single-cue probabilistic learning task. Group differences were evident for the multi-cue probabilistic learning task for associations that are weakly predictive (between 40 and 60%) but not when they are strongly predictive (10–20% or 80–90%). Computational modelling on the reinforcement learning task revealed that, as a group, autistic individuals had a higher learning rate than non-autistic individuals.

**Limitations:**

Due to the online nature of the study, we could not confirm the diagnosis of our autistic sample. The autistic participants were likely to have typical intelligence, and so our findings may not be generalisable to the entire autistic population. The learning tasks are constrained by a relatively small number of trials, and so it is unclear whether group differences will still be seen when given more trials.

**Conclusions:**

Autistic adults showed similar performance as non-autistic adults in learning associations by inferring a single cue or integrating multiple cues when the predictive strength was strong. However, non-autistic adults outperformed autistic adults when the predictive strength was weak, but only in the later phase. Autistic individuals were also more likely to incorporate prediction errors during decision making, which may explain their atypical performance on the weakly predictive associations. Our findings have implications for understanding differences in social cognition, which is often noisy and weakly predictive, among autistic individuals.

**Supplementary Information:**

The online version contains supplementary material available at 10.1186/s13229-025-00651-7.

## Introduction

Imagine you are from a culture that does not have the concept of ‘sadness’, and your new friend from a different culture is trying to demonstrate how sadness is expressed in their culture. They might show you a photograph of someone wailing and another photograph of someone weeping silently. In both photographs, you noticed that the expressers are shedding tears. Based on your limited experience with sad expressions, you might hypothesise that when someone is shedding tears, they are expressing sadness. You share your hypothesis with your new friend, and they show you a third photograph of someone holding a trophy while shedding tears, and your new friend tells you that person is expressing happiness and is crying ‘tears of joy’. You then update your hypothesis: when someone is shedding tears, they are expressing sadness *sometimes*. In other words, you update your belief that the presence of tears (the cue) is probabilistically associated with the expression of sadness (the outcome). Of course, learning to recognise emotion expressions in real life is a lot more complex than learning a simple one-to-one cue-outcome association. The example nonetheless demonstrates the idea that probabilistic learning, that is, learning cue-outcome associations that are probabilistic in nature, may be important for certain aspects of social cognition [1, 2].

Autistic individuals may find probabilistic learning to be challenging, according to recent theoretical models of autism that use Bayesian or predictive coding principles to understand characteristics of autism [3, 4]. Compared to non-autistic individuals, autistic individuals make less use of priors, that is, top-down knowledge acquired before the inference [5]. In the case of probabilistic learning, this may manifest as not taking advantage of similar past experiences with a particular cue to make an inference. Others have suggested that autistic individuals are more likely to incorporate feedback of the mismatch between top-down expectations and the outcome (‘prediction errors’) into their subsequent decision, even when the feedback should be ignored due to its unreliability or noise [6, 7]. The notion of prediction errors is also used in reinforcement learning models, which has been successful in accounting for learning behaviours in a probabilistic context [8]. Some claimed that autistic individuals have atypical learning of statistical regularities in the environment (‘statistical learning’) [9], such as the transitional probabilities of external events over time (for example, learning that Event B follows Event A 80% of the time). It has been further proposed that heterogeneity in autism may be partly due to individual differences in learning the strength of statistical regularities and temporal separation between events among autistic individuals [9].

While those models are theoretically sound, empirical evidence for them is mixed. In support of the models, some studies found group differences in learning transitional probabilities: after being presented with a sequence of stimuli, autistic children and adults were less likely to show behavioural and neural differences to probable vs. less probable sequences compared to non-autistic children and adults [10, 11]. Some evidence of group differences was also reported in studies using a probabilistic reversal learning task, which is commonly used to assess participants’ ability to learn cue-outcome contingencies in stable vs. volatile learning environment. In such a task, participants first learn the probabilistic associations of two cues and their outcomes over several trials (stable phase) and then the contingencies switch between the two cues such that the cue that was predictive of the reward is now less predictive (reversal phase). While no group differences between autistic and non-autistic participants were typically observed in the stable phase [12, 13], the reversal phase affected autistic participants more. Specifically, autistic participants tended to commit more perseverative errors (i.e., selecting the cue that was previously reinforced) as well as were less accurate and less likely to reach successful criterion threshold in the volatile phase [14–16]. When there were multiple reversal phases across the task, autistic participants were found to show smaller behavioural differences in expected vs. unexpected events compared to non-autistic participants [17]. Crucially, the authors demonstrated using computational modelling (i.e., using mathematical models to understand behaviour) that autistic individuals relative to non-autistic individuals tend to overestimate the volatility of the environment, compromising their ability to develop expectations even for highly predictive (i.e., 84% predictive) associations [17].

On the other hand, some studies have failed to find support for the Bayesian and predictive coding models of autism. Autistic and non-autistic children and adults learned repeated pattern sequences equally well [18–20] and showed similar performance on various statistical learning tasks [21]. A meta-analysis that examined statistical learning ability among autistic vs. non-autistic individuals found no evidence of group differences [23–25]. On the probabilistic reversal learning tasks, some studies reported that autistic individuals do not always show poorer performance after reversal [26]. These mixed findings may be due to methodological differences (e.g., task requirement and measurement) and heterogeneity of the participants (e.g., children or adult participants; whether autistic participants were matched with the non-autistic participants on cognitive or verbal abilities). It is thus difficult to pinpoint exactly why these mixed findings exist.

The past studies reviewed above revealed at least two gaps that limit the generalisability of the findings. Firstly, the previous studies often used simple cues (e.g., choosing between two coloured boxes) to learn their associations, which is often not the case in real life situations. Learners instead may have to infer a single target cue from a range of cues (e.g., presence of a smile despite variations in the other facial features usually expresses happiness) or integrate multiple cues (e.g., presence of furrowed brows, wide eyes, and loud and fast speech typically signals that the expresser is angry) to learn their associations with the outcome. While this has not been examined in detail, learning of such complex cue-outcome associations may be more challenging for autistic individuals for two reasons: autistic individuals’ tendency to (i) learn a reductive form of complex cue-outcome associations (the so-called ‘stimulus overselectivity’ phenomenon) [27]; and (ii) direct their focus to a small attention tunnel at the expense of processing stimuli outside the tunnel (‘monotropism’) [28]. One previous study partly examined this gap by investigating whether adults with varying levels of autistic traits would infer a target cue from multiple auditory cues (e.g., pitch, number of nonsense syllables, etc.) to learn cue-outcome associations, and the authors found no influence of autistic traits on such learning [29]. However, the task used in that study may have been too difficult, as it is reliant on one’s auditory memory to infer the target cue correctly, and there were only a small number of autistic participants in the sample.

In addition to the use of simple cues, most of the past studies also neglected to examine the learning of associations across a range of predictive strengths within the same sample. For instance, only one level of (typically high) predictive strength is examined in most probabilistic reversal learning tasks [13], even though most cue-outcomes associations in real life are weak given the complexity of the relationship particularly in social situations [1]. Some evidence suggests that group differences may be more pronounced for associations that are weakly predictive: in a task where participants learned the relationship between high vs. low tones and their associations with dots rotating clockwise vs. anticlockwise, both autistic and non-autistic participants showed improvement in learning the associations when the outcome contingency was 72.5% [24] but only the non-autistic participants did when the contingency was 62.5% [30]. The participants, however, were not the same across both studies, and so it remains to be seen whether the findings above were due to sampling differences or reflected genuine group differences.

The current study addressed both those gaps to examine several crucial aspects of probabilistic learning within the same sample of autistic and non-autistic adults. That is, we examine whether differential probabilistic learning performance between autistic and non-autistic individuals may be due to the complexity of the target cue (i.e., whether learners infer a single cue or integrate multiple cues to learn the association), the predictive strength of the association to be learned (i.e., whether the outcome contingency is weak vs. strong), and/or the incorporation of prediction error in their decision, as suggested by some models [6, 7]. Thus, participants completed three tasks in this study to examine whether autistic individuals show atypical probabilistic learning compared to non-autistic individuals, and if so, why this might be the case: (i) a single-cue probabilistic learning task, to examine whether they could learn to infer a single target cue from multiple cues to learn cue-outcome associations; (ii) a multi-cue probabilistic learning task, which compares learning associations of various predictive strengths by integrating multiple cues; and (iii) a reinforcement learning task, which requires learning the contingencies of two stimuli that have a probabilistic reinforcement schedule, from which we will use computational modelling to compare the model parameter on integrating prediction errors in decision making.

## Methods

### Participants

A total of 52 autistic adults (M_age_ = 29.08, SD_age_ = 6.75, Range = 18–43; Gender: Female *n* = 20, Male *n* = 27, Non-binary *n* = 5) and 52 non-autistic adults (M_age_ = 30.77, SD_age_ = 7.95, Range = 19–45; Gender: Female *n* = 24, Male *n* = 25, Non-binary *n* = 3), all of whom were recruited from Prolific, participated in the study and completed all three tasks in two sessions. The inclusion criteria for the autistic group were that they needed to have a confirmed diagnosis of autism (but due to the General Data Protection Regulation, GDPR, and the anonymity of online experiments, this could not be verified) and that they needed to have normal or corrected-to-normal vision. The inclusion criteria for the non-autistic group were that they must not have received a diagnosis of autism, have normal or corrected-to-normal vision, and that their Autism-Spectrum Quotient (AQ) score, which measures their levels of autistic traits, must be less than 32, the cut-off score recommended to distinguish autistic from non-autistic individuals [31]. The autistic group scored higher than the non-autistic group in AQ, as expected partly due to our inclusion criteria (*t*(102) = 12.15, *p* <.001), but the two groups did not differ in age (*t*(102) = 1.17, *p* =.245).

An additional five participants (Autistic *n* = 2; Non-autistic *n* = 3) completed Session 1 of the study but not Session 2; their data were excluded in the main analysis. One additional autistic participant completed both sessions but were ultimately excluded due to poor performance on the catch trials across the tasks (i.e., scoring less than 75% correct). Participants provided their informed consent at the start of the study and received monetary compensation for their participation. The study protocol was reviewed and approved by the University Research Ethics Committee (UREC) at the University of Reading.

### Materials & tasks

#### Single-cue probabilistic learning task

We adapted this task from a previous study [29], in which participants were presented with multiple auditory cues and they had to learn to infer which cue is the most predictive of an outcome. Visual cues were used in the current study instead. Participants were informed that they would learn to judge to which of two art periods each art piece belongs. Two different sets of art pieces were used, and each art piece consisted of four features (Set 1: dot colour, distribution, background colour, size; Set 2: shape, distribution, background colour, size). Unbeknown to the participants, only one feature in each set was predictive of the outcome (majority dot colour (black or white) in Set 1 and majority shape (x or o) in Set 2). No two stimuli were identical, and so participants needed to learn to abstract the features and learn the associative relationship between the feature and the outcome (i.e., art period). Each stimulus set was assigned to a condition: Deterministic (i.e., the target feature is 100% predictive of an art period) or Probabilistic (i.e., the target feature is 75% predictive of an art period). The assignment of stimulus set to condition was randomised for each participant, and participants completed both conditions in a randomised order.

**Fig. 1:**
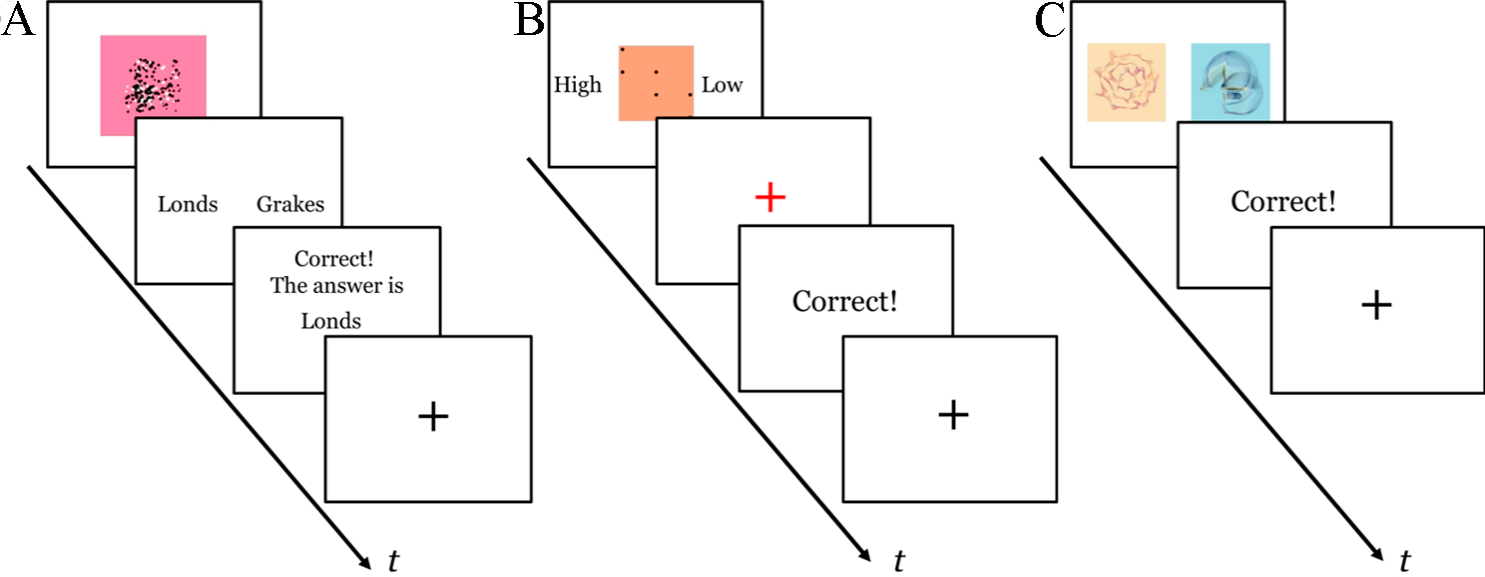
Trial structure for the (**A**) single-cue probabilistic learning task; (**B**) multi-cue probabilistic learning task; and (**C**) reinforcement learning task. In the single-cue probabilistic learning task (**A**), participants are first shown a stimulus for 1.5s. Then a response screen depicting two art periods (e.g., “Londs” and “Grakes”) is shown, during which participants have to respond within 2.5s. Feedback is then provided for 1s, followed by a fixation cross to signal the next trial. In the multi-cue probabilistic learning task (**B**), participants are first shown a stimulus, and they are asked to predict if the stimulus will receive a high or low rating within 5s. Then a red fixation cross to signal incoming feedback was presented for 1s, and the feedback was presented for 1.5s. The trial ends with a black fixation cross for a randomly jittered inter-trial interval (ITI) between 1.5s and 4.5s. In the reinforcement learning task (**C**), two stimuli are presented, and participants have to decide which has a higher value for a given year within 2.5s. This is followed by feedback for 1s, and then a fixation cross to signal the next trial

On every trial, participants were presented with the stimulus (an art piece) for 1.5s, and then a response screen appeared with the two art periods (e.g., “Londs” and “Grakes”), during which they had to respond within 2.5s. Feedback was provided for 1s immediately after their response (see Fig. 1a). There were 100 experimental trials in each condition, with 50 trials for each art period. For each condition, we divided the trials into the two halves to examine learning over time (Early vs. Late). To ensure participants were paying attention, in each condition, participants were presented with eight catch trials, in which the art period label was presented as the stimulus and participants were instructed to select that label. Each condition was preceded by six practice trials with uninformative feedback (i.e., they were presented with “#####” as feedback). Each condition took approximately 10 min to complete.

Similar to the previous study [29], we also presented participants with a control discrimination task after completing the main task to ensure that participants could perceptually discriminate the target feature (i.e., differentiate whether the majority of the dots were black/white or x/o). Participants were presented with the stimulus for 1.5s, just as they were in the main task, but they had unlimited time to respond whether the majority of the dots were black/white or x/o and they were not provided with any feedback. A subset of 10 stimuli for each category (e.g., majority black dots and majority white dots) were presented, resulting in a 20 trials per set. The presentation order for set was randomised. Two catch trials were included in each set, and prior to the start of each set, participants completed four practice trials. The entire control discrimination task took approximately 4 min to complete.

#### Multi-cue probabilistic learning task

We adapted the multi-cue probabilistic learning task from a previous study [32]. Unlike the single-cue probabilistic learning task, the multi-cue version requires learners to integrate multiple cues to determine their association with one of two outcomes. In our version, participants were asked to predict whether each art piece will receive a high or low rating from an art critic. They were specifically told that the art critic will base their rating on four binary cues: background colour (orange/purple), orientation (left/right), dot colour (black/white), and number of dots (3/6). The combination of all four cues resulted in 16 unique stimuli (or art pieces), with each stimulus associated with a probability that it will receive a high rating (see Table 1). On every trial, the probability is compared with a random number ranging from 0 to 1; if the probability is higher than the random number, then that art piece will receive a high rating for that trial. Thus, the relationship between each art piece and its rating are probabilistic in nature. To examine whether learning differs based on the predictive strength of the outcome, the high rating probability is divided into two conditions: unambiguous (those between 0.1 and 0.2 and 0.8–0.9) and ambiguous (those between 0.4 and 0.6). Across participants, cue assignment was randomised such that the importance of each cue would differ. For example, the cue ‘background colour’ may be assigned to the first cue (C1 in Table 1) for Participant A, but the same cue may be assigned to the second cue (C2) for Participant B. Given that the 16 stimuli are highly distinct, we anticipate that all participants should be able to discriminate the stimuli easily, and so we do not include a control discrimination task unlike in the single-cue probabilistic learning task.

The trial structure for this task is displayed in Fig. 1B. The stimulus was presented, and participants were required to make a response within 5s. Then a red fixation cross to signal incoming feedback was presented for 1s, and the feedback was presented for 1.5s. The trial ends with a black fixation cross for a randomly jittered inter-trial interval (ITI) between 1.5s and 4.5s. The 16 stimuli constituted a set, which were repeated eight times, for a total of 128 trials. The presentation order was blocked by and randomised within each set. The 128 trials were divided into two blocks (Early vs. Late) to examine learning over time. Eight catch trials were randomly presented throughout the task to ensure attentiveness, during which participants were presented with a label of “high” or “low” and were instructed to press the corresponding button. Prior to the experimental task, participants were given six practice trials with uninformative feedback (i.e., “#####” as feedback). The task took approximately 20 min to complete.

**Table 1:** Probability associated with high rating (p(high)) for the 16 stimuli, each made up of the four binary cues (C1, C2, C3 and C4), in the multi-cue probabilistic learning task. Based on their *p*(high), the stimuli are further divided into two conditions: unambiguous and ambiguous

Stimulus	C1	C2	C3	C4	*p*(High)	Condition
1	0	0	0	0	0.100	Unambiguous
2	0	0	0	1	0.133	Unambiguous
3	0	0	1	0	0.166	Unambiguous
4	0	1	0	0	0.200	Unambiguous
5	1	0	0	0	0.400	Ambiguous
6	1	0	0	1	0.429	Ambiguous
7	1	0	1	0	0.458	Ambiguous
8	1	1	0	0	0.487	Ambiguous
9	1	1	0	1	0.516	Ambiguous
10	1	1	1	0	0.545	Ambiguous
11	0	0	1	1	0.574	Ambiguous
12	1	0	1	1	0.600	Ambiguous
13	0	1	1	0	0.800	Unambiguous
14	0	1	0	1	0.833	Unambiguous
15	0	1	1	1	0.866	Unambiguous
16	1	1	1	1	0.900	Unambiguous

#### Reinforcement learning task

The reinforcement learning task in the present study is similar to a two-armed bandit task [33]. Participants were told to judge which of two art pieces has a higher value for a given year, with the same two stimuli were presented on every trial (see Fig. 1C), within 2.5s. One of the stimuli was associated with a higher value 70% of the time, which was determined randomly at the start of the task for each participant. Immediately after response, feedback was displayed for 1s, and then a fixation cross for 0.5s before the next trial began. The reward contingencies stayed constant across the experiment and there were no reversals. There were 20 experimental trials plus two catch trials randomly interspersed among the experimental trials. Four practice trials preceded the task, with uninformative feedback. The task took about 2 min to complete.

### Procedure

Participants first completed a screening questionnaire, in which they answered questions about their demographic information (including whether they have received a clinical diagnosis of autism) and completed the Autism Spectrum Quotient [31]. Eligible participants were then invited to the two-session study hosted on the Gorilla platform [34]. We decided to split the study into two sessions of less than 30 min each rather than a single session of just under an hour to prevent fatigue and boredom among participants and therefore minimise dropout rates in line with good practice suggestions for online research [35]. In the first session, they completed the single-cue probabilistic learning task and the reinforcement learning task. In the second session, to which they were invited only after completing the first session, participants completed the multi-cue probabilistic learning task. On top of receiving monetary reimbursement for each session, a completion monetary bonus was offered to participants who successfully completed both sessions.

### Data analysis

Data analysis was conducted in R (version: 4.1.2) [36].

#### Single-cue probabilistic learning task

For the control task, we calculated d-prime (d’) scores according to the signal detection theory where d’ = z(Hit)– z(False Alarm), separately for black/white and x/o discrimination for each participant. Extreme values of 0 and 1 for Hit and False Alarm rates were adjusted upwards and downwards by 0.01, respectively [37]. Groups were compared on their d’ scores using independent *t*-tests, and above-chance performance was determined by comparing their d’ scores against 0.

For the main probabilistic learning task, we fitted a binomial mixed effects model, with a binary dependent variable Correct (correct/incorrect, with ‘correct’ defined as the most probable outcome) using the *glmer()* function from the *lme4* package [38]. As fixed effects, we entered Phase (Early vs. Late), Condition (Deterministic vs. Probabilistic), and Group (Autistic vs. Non-Autistic) and all the possible interactions. We also entered the d’ scores of the control task as fixed effects, to account for perceptual differences among participants. All categorical predictors were effect-coded whereas continuous predictors were mean-centred. As random effects, random by-participant and by-item intercepts and random by-participant slope for Phase and Condition as well as random by-item slope for Group was included. Statistical significance of each fixed effect was determined using the *Anova()* function from the *car* package [39]. Pairwise comparisons were conducted using the *emmeans* package [40].

#### Multi-cue probabilistic learning task

Similar to the single-cue probabilistic learning task, we fitted a binomial mixed effects model, with Correct as the binary dependent variable (‘correct’ defined as the most probable outcome) using the *glmer()* function from the *lme4* package [38]. We entered the following as fixed effects: Phase (Early vs. Late), Condition (Ambiguous vs. Unambiguous), Group (Autistic vs. Non-Autistic) and all the possible interactions. Categorical predictors were effect-coded. *p*-values for the predictors were determined using the *Anova()* function from the *car* package [39], and subsequent pairwise comparisons were conducted using the *emmeans* package [40].

#### Reinforcement learning task

We use the *hBayesDM* package [41] to fit three different reinforcement learning models. Reinforcement learning models are widely used in social neuroscience and decision making [2] and are suitable given that learners use feedback to guide decision making through the modification of the expected reward.

The first model we fitted was a simple Rescorla-Wagner model [42], which is expressed by the equations below:

$$\text{Value}\:\text{update}:\:{V}_{t}={V}_{t-1}+\:{\alpha\:\:\delta\:}_{t-1}$$

$$\text{Prediction}\:\text{error}:\:{\delta\:}_{t-1}={R}_{t-1}-\:{V}_{t-1}$$

where the expected value of a chosen option in the current trial (V_t_) is informed by the expected value of the previous trial (V_t−1_) and the prediction error (δ, or the difference between the reward (R) and the expected value) of the previous trial, scaled by the learning rate (0 < α < 1). The learning rate thus dictates how much of the prediction error should be considered in the value update: the higher the learning rate, the more the prediction error is weighted. This model was implemented using the *bandit2arm_delta()* function.

The second reinforcement learning model we fitted was a positive/negative Rescorla-Wagner model [43], a variant of the simple Rescorla-Wagner model, and is formulated as below:

$$\:\left\{\begin{array}{c}{\:V}_{t}={V}_{t-1}+\:{{\alpha\:}^{+}\delta\:}_{t-1},\:\:\:\:\:if\:\:\:\:{\delta\:}_{t-1}\ge\:0\\\:{\:V}_{t}={V}_{t-1}+\:{{\alpha\:}^{-}\delta\:}_{t-1},\:\:\:\:\:if\:\:\:\:{\delta\:}_{t-1}<0\end{array}\right.$$

The positive/negative Rescorla-Wagner model is similar to the simple version, with the exception that there are two separate learning rates: one that scales positive (including zero-difference) prediction errors (α^+^) and one that scales negative prediction errors (α^−^). A positive prediction error would occur if the expected value were smaller than the reward (e.g., when one does not expect a reward but receives one) whereas a negative prediction error would occur if the expected value were larger than the reward (e.g., when one does expect a reward but does not receive one). This model thus considers that learning may be different when one receives rewards or punishments. We used the *prl_rp()* function to implement this model.

The third and final model we fitted was a counterfactual Rescorla-Wagner model [44], another variant of the simple Rescorla-Wagner model. The model is formulated as below:

$$\begin{aligned}\text{Value}\:\text{update}:\:&{V}_{t}^{c}={V}_{t-1}^{c}+\:\alpha\:\:{\delta\:}_{t-1}^{c}\\&{V}_{t}^{nc}={V}_{t-1}^{nc}+\:\alpha\:\:{\delta\:}_{t-1}^{nc}\end{aligned}$$

$$\begin{aligned}\text{Prediction}\:\text{error}:&\:{\delta\:}_{t-1}^{c}=\:{R}_{t-1}-\:{V}_{t-1}^{c}\:\\&{\delta\:}_{t-1}^{nc}=\:{-R}_{t-1}-\:{V}_{t-1}^{nc}\end{aligned}$$

Whereas the simple Rescorla-Wagner model only updates the value for the chosen option, the counterfactual Rescorla-Wagner model does value-updating for the chosen option (V^c^) *and* the unchosen option (V^nc^). Given that the task has anti-correlated choice values (e.g., if the chosen option is rewarded, then the unchosen option is not rewarded), learners can thus learn from the counterfactual outcome. This model was implemented using the *prl_fictitious()* function.

The three reinforcement learning models were fitted to each participant’s trial-by-trial data. Timed out trials were excluded in the analysis. For each group, we then used the leave-one-out cross-validation information criterion (LOOIC) to find the best fitting model where the lower the LOOIC, the better the model fit. We then compared the groups on the learning rates (α) obtained from the best fitting model to examine whether autistic individuals have atypical weighting on the predictions errors relative to non-autistic individuals.

## Results

Descriptive statistics for all three tasks along with results of the one-sample *t*-tests to determine above chance performance for the single-cue and multi-cue tasks are presented in Table 2.

**Table 2:** Mean (standard deviation in parathesis) performance for the single-cue probabilistic learning task and the multi-cue probabilistic learning task as well as mean learning rate (standard deviation in parenthesis) for the reinforcement learning task by group. Asterisks indicate above chance performance (50%) for the single-cue and multi-cue learning tasks based on one-sample t-tests (this was not conducted for the reinforcement learning task as learning rate cannot be determined by chance)

		Autistic (*n* = 52)	Non-autistic (*n* = 52)
*Single-cue task*			
Deterministic	Early	0.63 (0.19)***	0.62 (0.19)***
	Late	0.72 (0.24)***	0.70 (0.24)***
Probabilistic	Early	0.58 (0.15)***	0.58 (0.16)**
	Late	0.61 (0.18)***	0.61 (0.18)***
*Multi-cue task*			
Unambiguous	Early	0.61 (0.12)***	0.64 (0.13)***
	Late	0.63 (0.13)***	0.64 (0.12)***
Ambiguous	Early	0.58 (0.12)***	0.56 (0.11)***
	Late	0.55 (0.10)**	0.59 (0.10)***
*Reinforcement learning task*			
	Learning Rate	0.39 (0.05)	0.34 (0.06)

Note: *p <.05, **p <.01, ***p <.001

### Single-cue probabilistic learning task

We first compared the groups on their discrimination of the two target cues in the single-cue probabilistic learning task. Independent *t*-tests on the d’ scores revealed that the two groups did not differ in the black/white discrimination task (*t*(102) = 0, *p* = 1) but autistic participants had higher d’ scores than non-autistic participants on the x/o discrimination task (*t*(102) = 2.37, *p* =.020). The two groups, crucially, could reliably discriminate the target features, with their d’ scores well above chance (Autistic: black/white, *t*(51) = 104.39, *p* <.001; x/o, *t*(51) = 38.29, *p* <.001. Non-autistic: black/white, *t*(51) = 87.28, *p* <.001; x/o, *t*(51) = 31.77, *p* <.001).

**Fig. 2:**
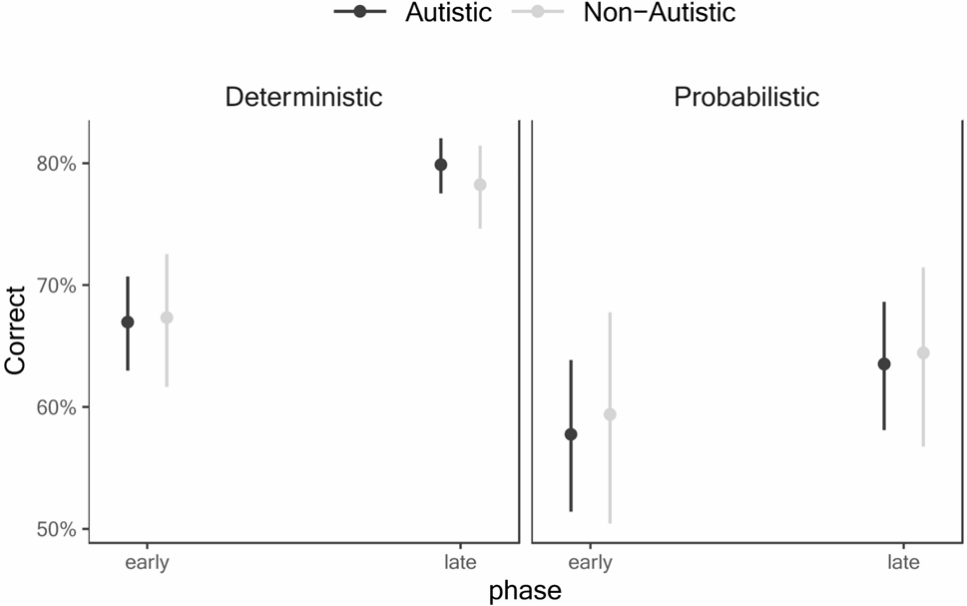
Mean proportion correct for the single-cue probabilistic learning task as a function of group, phase, and condition. Error bars represent 95% confidence intervals

For the main single-cue probabilistic learning task, participants showed above chance performance in all conditions and phases suggesting that learning has occurred even in the Early phase (see Table 2). Results on the mixed effects model are displayed in Table 3. There was a main effect of Discrimination of x/o on accuracy of the single-cue probabilistic learning task such that higher d’ scores were related to better performance overall (χ^2^(1) = 6.18, *p* =.013). There were also main effects of Phase (χ^2^(1) = 48.54, *p* <.001) and Condition (χ^2^(1) = 21.87, *p* <.001), which were qualified by a Phase × Condition interaction (χ^2^(1) = 36.59, *p* <.001). Pairwise comparisons revealed that the performance was significantly better on the Deterministic condition than the Probabilistic condition, but the difference between the conditions was larger in the Late phase (*z* = 6.02, *p* <.001) than in the Early phase (*z* = 2.99, *p* =.003), as shown in Fig. 2. Comparison between Early vs. Late phases for each Condition showed that although participants showed significant improvement, the improvement was larger for the Deterministic condition (*z* = 8.74, *p* <.001) than for the Probabilistic condition (*z* = 3.43, *p* =.001). Importantly, there were no significant effects or interactions involving Group, suggesting that performance among autistic and non-autistic participants was similar in the single-cue probabilistic learning task, and the improvement was similar across both groups.

**Table 3:** Output of the mixed effects model for the single-cue probabilistic learning task

	χ^2^	df	*p*
Intercept	69.00	1	< 0.001
Phase	48.54	1	< 0.001
Condition	21.87	1	< 0.001
Group	0.00	1	0.973
d’ black/white	0.09	1	0.762
d’ x/o	6.18	1	0.013
Phase × Condition	36.59	1	< 0.001
Phase × Group	0.35	1	0.553
Condition × Group	0.16	1	0.693
Phase × Condition × Group	0.48	1	0.491

Note: Phase: Early vs. Late; Condition: Deterministic vs. Probabilistic; Group: Autistic vs. Non-autistic. The final model: Correct ~ Phase*Condition*Group + d’ black/white + d’ x/o + (1 + Condition + Phase|participant) + (1 + Group|image)

### Multi-cue probabilistic learning task

**Fig. 3:**
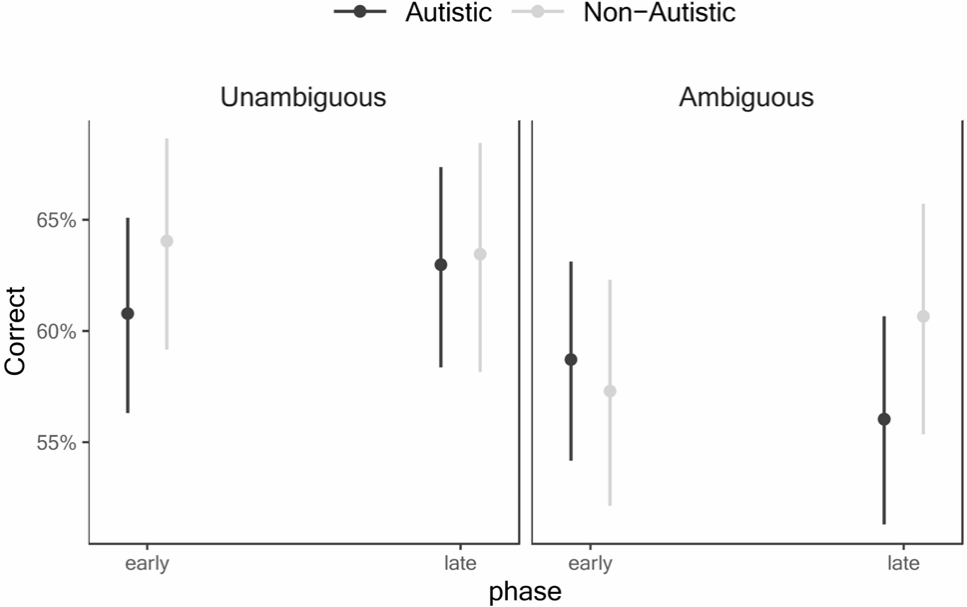
Mean proportion correct for the multi-cue probabilistic learning task as a function of Group, Phase, and Condition. Error bars represent 95% confidence intervals

Participants showed above chance performance in all conditions and phases suggesting that learning has occurred even in the Early phase (see Table 2). Table 4 shows the output of the mixed effects model for the multi-cue probabilistic learning task. There was a significant effect of Condition (χ^2^(1) = 16.49, *p* <.001), and a significant interaction between Group × Phase × Condition (χ^2^(1) = 6.37, *p* =.012). Figure 3 displays the three-way interaction. Pairwise comparisons between Phases for each Group and Condition revealed only a marginally significant improvement from Early vs. Late phases for non-autistic participants in the Ambiguous condition (*z* = 1.83, *p* =.067). Pairwise comparisons between Groups, however, revealed that for the Unambiguous condition, performance was similar between autistic and non-autistic participants in both the Early (*z* = 1.16, *p* =.246) and Late (*z* = 0.17, *p* =.862) phases, whereas for the Ambiguous condition, there was no group difference in the Early phase (*z* = 0.60, *p* =.552) but non-autistic participants had higher performance than autistic participants in the Late phase (*z* = 2.09, *p* =.037). Thus, taken together with the results from the one-sample *t*-tests, this suggests that while participants learned during the Early phase itself, they did not demonstrate significant improvement during the task from Early to Late phases. However, group differences do emerge in the Late phase only for the Ambiguous condition.

**Table 4:** Output of the mixed effects model for the multi-cue probabilistic learning task

	χ^2^	df	*p*
Intercept	20.51	1	< 0.001
Group	0.80	1	0.370
Phase	0.36	1	0.550
Condition	16.49	1	< 0.001
Group × Phase	0.64	1	0.423
Group × Condition	0.02	1	0.876
Phase × Condition	0.07	1	0.791
Group × Phase × Condition	6.37	1	0.012

Note: Group: Autistic vs. Non-autistic; Phase: Early vs. Late; Condition: Ambiguous vs. Unambiguous. The final model: Correct ~ Group*Phase*Condition + (1 + Condition + Phase|participant) + (1 + Group|image)

### Reinforcement learning task

**Fig. 4:**
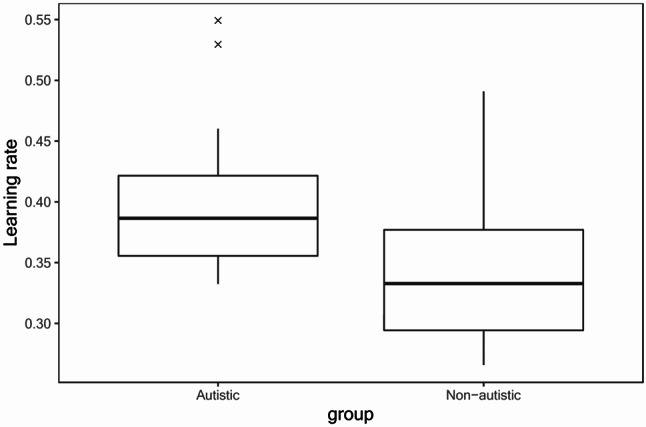
Boxplots of the learning rates obtained from the counterfactual model by group

Table 5 shows the LOOIC values for each of the three reinforcement learning models– simple Rescorla-Wagner, positive/negative Rescorla-Wagner, and counterfactual Rescorla-Wagner– by group. For both autistic and non-autistic groups, model comparisons showed that the counterfactual model had the best fit (i.e., the lowest LOOIC value). We then compared the groups on their learning rates obtained from the counterfactual model using an independent *t*-test. We found that the learning rate for the autistic participants as a group were significantly higher than that for the non-autistic participants (*t*(102) = 4.92, *p* <.001), as shown in Fig. 4. The group difference remains significant even after excluding the two outliers among autistic participants (*t*(100) = 4.64, *p* <.001).

**Table 5:** Comparison of model fit using LOOIC values for the three reinforcement learning models by group

Group	Simple	Positive/Negative	Counterfactual
Autistic	1023.29	943.58	907.32
Non-autistic	1021.73	973.98	970.17

## Discussion

Proponents of Bayesian and predictive coding models of autism theorised that autistic individuals may find probabilistic learning, that is, learning cue-outcome associations that have some degree of noise, to be more challenging than non-autistic individuals [5–7, 9, 45]. Empirical evidence for this, however, is mixed. To clarify the mixed findings and to better understand whether probabilistic learning is indeed atypical among autistic individuals, we examined in the present study three crucial aspects of probabilistic learning—(i) inferring or integrating target cues, (ii) learning from a range of predictive strengths, and (iii) incorporation of prediction error—within the same sample to determine whether group differences exist in any (or all) of the aspects.

Extending previous work that used simpler stimuli [12, 13, 24, 25], we found that autistic adults showed comparable performance as non-autistic adults in inferring either a single cue from multiple cues or integrating multiple cues to learn associations that are at least 70% predictive. On inferring from a single cue, our finding is similar to that of a previous study that used auditory stimuli [29], suggesting that there are no group differences regardless of stimulus modality. We are not aware of any studies that have examined group differences in learning associations that require integrating multiple cues, but we reasoned that this may be more challenging for autistic participants based on certain characteristics of autism. Two examples of such characteristics are stimulus overselectivity, or the tendency to associate one aspect of a complex cue to an outcome [27], and monotropism, or the tendency to only focus on one thing at a time [28]. Our findings suggest that, despite those characteristics, autistic individuals showed comparable learning by integrating multiple cues as non-autistic individuals when the associations are strongly predictive. Note, though, that the multiple cues to be integrated in this study are within the same modality, and thus it is not clear whether this would be generalisable when the cues are from different modalities. Indeed, autistic individuals have been reported to show atypical multisensory integration (e.g., audio and visual cues) [46], and so it remains to be seen whether there are any group differences when learning associations from integrating multiple cues across different modalities.

While no group differences were found when associations are strongly predictive, we found that autistic participants showed lower performance than non-autistic participants when they had to integrate multiple cues to learn weakly predictive associations (i.e., that are 40–60% predictive) towards the latter end of the learning task. Additionally, in a separate task through computational modelling, we found that autistic individuals had higher learning rates—that is, autistic individuals were more likely incorporate prediction errors during decision making—than non-autistic individuals. Our finding of higher learning rates among autistic individuals is consistent with the High, Inflexible Precision of Prediction Error in Autism (HIPPEA) hypothesis [7], in which it is stated that autistic individuals may find it more difficult to ignore noisy or unreliable prediction errors than non-autistic individuals. We speculate that having higher learning rates may also explain autistic individuals’ atypical performance on learning associations with weak predictive strength, which would have noisier errors than associations with strong predictive strength. One study demonstrated computationally that individuals with higher learning rates are more reliant on the most immediate past trials to inform value updating on the current trial [2]. In other words, those with higher learning rates would be placing less importance on older than recent past trials to inform their current decision. Thus, in a situation where there is frequent noisy, unreliable feedback (i.e., when learning associations with weak predictive strength), those with higher learning rates essentially sample from a smaller prior window of past trials to inform their decision compared to those with lower learning rates. The small window is likely to have more variance or dissimilar outcomes among the past trials given the weak predictive strength, leading to a less ‘accurate’ or ‘complete’ estimate relative to those who sample from a larger window of past trials (i.e., those with lower learning rates). The difference among those with high vs. low learning rates is less apparent when learning associations with strong predictive strength because there would be less variance in the past trials sampled, regardless of the window size, as the feedback would be more consistent. Due to task and methodology differences, we are unable to compare how learning rates obtained in the reinforcement learning task is directly involved in learning associations of various predictive strengths, and so this remains a speculation, which should be examined in future research.

If autistic individuals are less likely to ignore noisy prediction errors and find learning weakly predictive associations to be more challenging as found in the present study, might this explain autistic individuals’ differences in social cognition? Some have conceptualised social cognitive processes in terms of a cue integration framework [1]—for example, to recognise someone’s emotion, one needs to integrate many social cues across different modalities and contexts. These social cues and contexts are often complex and sometimes contradictory, and so the predictive strength of the associations between the cues and the emotion will likely be weak. Given the often-reported atypical performance in emotion recognition among autistic individuals compared to non-autistic individuals [47–50], it is thus tempting to surmise that autistic individuals’ emotion perception (and potentially any atypical social cognitive processes) may be partly related to their ability to ignore noisy prediction errors and learn weakly predictive associations. Whether there is such a direct link should be determined in future research to fully understand the mechanisms underlying the differences in social cognition among autistic individuals.

### Limitations

There are several limitations in this study that should be noted. The first concerns the autistic sample. Due to the nature of the online platform used in this study (i.e., Prolific), we could only recruit adults, and we were unable to confirm whether the autistic participants have truly received a clinical diagnosis of autism. While verification is not possible for this study, we are somewhat reassured by the fact that the majority of participants on Prolific are generally quite honest in their response (i.e., they do not claim a reward for something that they did not do) [51]. Moreover, the adults on Prolific are likely to be well-educated and have typical intelligence—one study found that the median response for the highest level of education attained among Prolific respondents was a bachelor’s degree [52]. So, it is unclear whether our findings would be generalisable to the entire autistic population.

Another limitation concerns the task– while we found group differences in integrating multiple cues with low predictive strength (i.e., in the multi-cue probabilistic learning task), we did not examine whether similar group differences would be found in the weakly predictive associations in single-cue probabilistic learning task, in which participants infer a single target cue from many cues. If indeed the reason for group difference in learning associations with low predictive strength is related to autistic individuals’ higher precision in their prediction errors, then we can expect that it is likely to be the case.

The lack of a significant effect of phase for the non-autistic participants in the multi-cue task is indeed puzzling, particularly since there is a significant group difference in the later phase only for the ambiguous associations (i.e., the three-way interaction). Even though both groups showed above-chance performance even in the Early phase, suggesting that they learned to some degree, they did not show significant improvement from the first half to the second half of the task. This may be the a result of an arbitrary division of trials into phases coupled with the relatively small number of trials. Indeed, participants have to learn 16 associations, with only 8 repetitions per association in the multi-cue task. It may be that significant improvement may only be seen with more trials given the difficult nature of the task. We explored the data using Generalised Additive Mixed Models (GAMM) to examine whether the trajectories between the groups were different, particularly for the ambiguous condition in the multi-cue task (see Additional Material 1). In summary, we found that, similar to the results reported in the manuscript, the overall performance among the non-autistic group was not significantly different from the autistic group for the ambiguous condition in the multi-cue task. However, their trajectories *were* significantly different: whereas the non-autistic group appears to be improving over time, the autistic group appears to be performing worse. Towards the end of the task, performance among both groups diverged, though not quite completely. This supports the idea that with more trials, a clearer group difference may emerge. It is of course also possible that autistic individuals may eventually ‘catch up’ with more trials, revealing similar performance as non-autistic individuals. This could be examined in future research.

The final limitation concerns the interpretation of the single-cue task findings. In the single-cue task, autistic individuals discriminated the x/o stimuli better than non-autistic individuals (though both groups performed above chance), and probabilistic learning performance was related to perceptual ability—that is, how well one discriminated the stimuli (x/o). While participants’ perceptual ability was accounted for in the model to minimize its impact, this may have obscured any potential group effects in the model. Our findings in the multi-cue task somewhat address this issue: the stimuli presented in the multi-cue task are easily discriminable, and we found no group difference when the predictive strength of the stimulus is strong (i.e., at least 80% predictive), similar to the single-cue task (i.e., either 75% or 100% predictive). Thus, it is unlikely that the lack of a group difference in the single-cue task is entirely due to group effects being obscured by their perceptual ability. Nonetheless, future studies should attempt to replicate the single-cue task using more easily discriminable stimuli to prevent any potential confounding effects of perceptual ability on probabilistic learning.

## Conclusion

This study found that autistic adults showed similar performance as non-autistic adults in learning associations by inferring a single cue or integrating multiple cues when the predictive strength was strong. However, non-autistic adults outperformed autistic adults when the predictive strength was weak, but only in the later phase. Given the relatively small number of trials for an arguably difficult task, this finding needs to be confirmed with better methodological refinement. We also found that autistic individuals were more inclined to incorporate prediction errors during decision making, which may explain their atypical performance on learning weakly predictive associations. Overall, then, this suggests that atypical probabilistic learning is observed among autistic individuals when learning associations that are weakly predictive, presumably due to their difficulty ignoring noisy or unreliable feedback. Our findings have implications for understanding differences in social cognition, which is often noisy and weakly predictive, among autistic individuals.

## Electronic supplementary material

Below is the link to the electronic supplementary material.

Supplementary Material 1

## Data Availability

The datasets generated and/or analysed during the current study are available in the OSF repository, https://osf.io/7z5p4/.

## References

[1] Zaki J. Cue integration: a common framework for social cognition and physical perception. Perspect Psychol Sci. 2013;8(3):296–312. 26172972 10.1177/1745691613475454

[2] Zhang L, Lengersdorff L, Mikus N, Gläscher J, Lamm C. Using reinforcement learning models in social neuroscience: frameworks, pitfalls and suggestions of best practices. Soc Cogn Affect Neurosci. 2020;15(6):695–707. 32608484 10.1093/scan/nsaa089 PMC7393303

[3] Haker H, Schneebeli M, Stephan KE. Can bayesian theories of autism spectrum disorder help improve clinical practice? Front Psychiatry. 2016;7(107):1–17. 27378955 10.3389/fpsyt.2016.00107 PMC4911361

[4] Palmer CJ, Lawson RP, Hohwy J. Bayesian approaches to autism: towards volatility, action, and behavior. Psychol Bull. 2017;143(5):521–42. 28333493 10.1037/bul0000097

[5] Pellicano E, Burr D. When the world becomes ‘too real’: a bayesian explanation of autistic perception. Trends Cogn Sci. 2012;16(10):504–10. 22959875 10.1016/j.tics.2012.08.009

[6] Lawson RP, Rees G, Friston KJ. An aberrant precision account of autism. Front Hum Neurosci. 2014;8(May):1–10. 24860482 10.3389/fnhum.2014.00302 PMC4030191

[7] Van de Cruys S, Evers K, Van der Hallen R, Van Eylen L, Boets B, De-Wit L, et al. Precise minds in uncertain worlds: predictive coding in autism. Psychol Rev. 2014;121(4):649–75. 25347312 10.1037/a0037665

[8] Friston K. Does predictive coding have a future? Nat Neurosci. 2018;21(8):1019–21. 30038278 10.1038/s41593-018-0200-7

[9] Sinha P, Kjelgaard MM, Gandhi TK, Tsourides K, Cardinaux AL, Pantazis D, et al. Autism as a disorder of prediction. Proc Natl Acad Sci. 2014;111(42):15220–5. 25288765 10.1073/pnas.1416797111 PMC4210351

[10] Fogelson N, Li L, Diaz-Brage P, Amatriain-Fernandez S, Valle-Inclan F. Altered predictive contextual processing of emotional faces versus abstract stimuli in adults with Autism Spectrum Disorder. Clin Neurophysiol. 2019;130(6):963–75. 31003115 10.1016/j.clinph.2019.03.031

[11] Wagley N, Lajiness-O’Neill R, Hay JSF, Bowyer SM, Ugolini M, Kovelman I, et al. Predictive processing during a naturalistic statistical learning task in ASD. eNeuro. 2020. 10.1523/ENEURO.0069-19.2020 PMC7729300 33199412

[12] Costescu CA, Vanderborght B, David DO. Reversal learning task in children with autism spectrum disorder: a robot-based approach. J Autism Dev Disord. 2015;45(11):3715–25. 25479815 10.1007/s10803-014-2319-z

[13] D’Cruz AM, Ragozzino ME, Mosconi MW, Shrestha S, Cook EH, Sweeney JA. Reduced behavioral flexibility in autism spectrum disorders. Neuropsychology. 2013;27(2):152–60. 23527643 10.1037/a0031721 PMC3740947

[14] Crawley D, Zhang L, Jones EJH, Ahmad J, Oakley B, San José Cáceres A, et al. F Ramus editor 2020 Modeling flexible behavior in childhood to adulthood shows age-dependent learning mechanisms and less optimal learning in autism in each age group. PLOS Biol 18 10 e3000908. 33108370 10.1371/journal.pbio.3000908 PMC7591042

[15] Robic S, Sonié S, Fonlupt P, Henaff MA, Touil N, Coricelli G, et al. Decision-making in a changing world: a study in autism spectrum disorders. J Autism Dev Disord. 2015;45(6):1603–13. 25433404 10.1007/s10803-014-2311-7

[16] South M, Newton T, Chamberlain PD. Delayed reversal learning and association with repetitive behavior in autism spectrum disorders. Autism Res. 2012;5(6):398–406. 23097376 10.1002/aur.1255

[17] Lawson RP, Mathys C, Rees G. Adults with autism overestimate the volatility of the sensory environment. Nat Neurosci. 2017;20(9):1293–9. 28758996 10.1038/nn.4615 PMC5578436

[18] Nemeth D, Janacsek K, Balogh V, Londe Z, Mingesz R, Fazekas M, et al. Learning in autism: implicitly superb. PLoS ONE. 2010;5(7). 10.1371/journal.pone.0011731 PMC2908691 20661300

[19] Zwart FS, Vissers CTWM, Kessels RPC, Maes JHR. Implicit learning seems to come naturally for children with autism, but not for children with specific language impairment: evidence from behavioral and ERP data. Autism Res. 2018;11(7):1050–61. 29676529 10.1002/aur.1954 PMC6120494

[20] Zwart FS, Vissers CTWM, Maes JHR. The association between sequence learning on the serial reaction time task and social impairments in autism. J Autism Dev Disord. 2018;1–9. 10.1007/s10803-018-3529-6 PMC6061016 29524017

[21] Brown J, Aczel B, Jiménez L, Kaufman SB, Grant KP. Intact implicit learning in autism spectrum conditions. Q J Exp Psychol. 2010;63(9):1789–812. 10.1080/17470210903536910 20204919

[22] Obeid R, Brooks PJ, Powers KL, Gillespie-Lynch K, Lum JAG. Statistical learning in specific language impairment and autism spectrum disorder: a meta-analysis. Front Psychol. 2016;7(AUG):1–18. 27602006 10.3389/fpsyg.2016.01245 PMC4993848

[23] Retzler C, Boehm U, Cai J, Cochrane A, Manning C. Prior information use and response caution in perceptual decision-making: no evidence for a relationship with autistic-like traits. Q J Exp Psychol. 2021;17470218211019939. 10.1177/17470218211019939 PMC8450985 33998332

[24] Sapey-Triomphe LA, Temmerman J, Puts NAJ, Wagemans J. Prediction learning in adults with autism and its molecular correlates. Mol Autism. 2021;12(1):64. 34615532 10.1186/s13229-021-00470-6 PMC8493731

[25] Solomon M, Smith AC, Frank MJ, Ly S, Carter CS. Probabilistic reinforcement learning in adults with autism spectrum disorders. Autism Res. 2011;4(2):109–20. 21425243 10.1002/aur.177 PMC5538882

[26] Manning C, Kilner J, Neil L, Karaminis T, Pellicano E. Children on the autism spectrum update their behaviour in response to a volatile environment. Dev Sci. 2017;20(5):1–13. 10.1111/desc.12435 PMC5600083 27496590

[27] Ploog BO. Stimulus overselectivity four decades later: a review of the literature and its implications for current research in Autism Spectrum Disorder. J Autism Dev Disord. 2010;40(11):1332–49. 20238154 10.1007/s10803-010-0990-2

[28] Murray D, Lesser M, Lawson W. Attention, monotropism and the diagnostic criteria for autism. Autism. 2005;9(2):139–56. 15857859 10.1177/1362361305051398

[29] Ong JH, Liu F. Probabilistic learning of cue-outcome associations is not influenced by autistic traits. J Autism Dev Disord. 2023;53:4047–59. 35951205 10.1007/s10803-022-05690-0 PMC9366807

[30] Sapey-Triomphe LA, Weilnhammer VA, Wagemans J. Associative learning under uncertainty in adults with autism: intact learning of the cue-outcome contingency, but slower updating of priors. Autism. 2021;13623613211045026. 10.1177/13623613211045026 34533061

[31] Baron-Cohen S, Wheelwright S, Skinner R, Martin J, Clubley E. The autism-spectrum quotient (AQ): evidence from Aperger syndrome/high-functioning autism, males, and females, scientists and mathematicians. J Autism Dev Disord. 2001;31(1):5–17. 11439754 10.1023/a:1005653411471

[32] Newell BR, Weston NJ, Tunney RJ, Shanks DR. The effectiveness of feedback in multiple-cue probability learning. Q J Exp Psychol. 2009;62(5):890–908. 10.1080/17470210802351411 18932062

[33] McDougle SD, Boggess MJ, Crossley MJ, Parvin D, Ivry RB, Taylor JA. Credit assignment in movement-dependent reinforcement learning. Proc Natl Acad Sci. 2016;113(24):6797–802. 27247404 10.1073/pnas.1523669113 PMC4914179

[34] Anwyl-Irvine AL, Massonnié J, Flitton A, Kirkham N, Evershed JK. Gorilla in our midst: an online behavioral experiment builder. Behav Res Methods. 2020;52(1):388–407. 31016684 10.3758/s13428-019-01237-x PMC7005094

[35] Gagné N, Franzen L. How to Run Behavioural Experiments Online: Best Practice Suggestions for Cognitive Psychology and Neuroscience. Swiss Psychol Open Off J Swiss Psychol Soc [Internet]. 2023 Jan 4 [cited 2024 Nov 4];3(1). Available from: https://swisspsychologyopen.com/articles/10.5334/spo.34

[36] R Core Team. R: a language and environment for statistical computing. Vienna, Austria: R Foundation for Statistical Computing; 2021.

[37] Macmillan NA, Creelman CD. Detection theory: a user’s guide. 2nd ed. Lawrence Erlbaum Associates; 2005.

[38] Bates D, Maechler M, Bolker B, Walker S. Fitting linear mixed-effects models using lme4. J Stat Softw. 2015;67(1):1–48.

[39] Fox J, Weisberg S, An R. Companion to Applied Regression [Internet]. 3rd ed. Thousand Oaks, CA: Sage; 2019. Available from: https://socialsciences.mcmaster.ca/jfox/Books/Companion/

[40] Lenth RV. emmeans: Estimated Marginal Means, aka Least-Squares Means [Internet]. 2019. Available from: https://cran.r-project.org/package=emmeans

[41] Ahn WY, Haines N, Zhang L. Revealing neurocomputational mechanisms of reinforcement learning and decision-making with the hBayesDM package. Comput Psychiatry. 2017;1(0):24–57. 10.1162/CPSY_a_00002 PMC5869013 29601060

[42] Rescorla RA, Wagner AR. A theory of Pavlovian conditioning: Variations in the effectiveness of reinforcement and nonreinforcement. In: Black AH, Procasy WF, editors. Classical conditioning II: Current research and theory [Internet]. New York: Appleton-Century-Crofts; 1972. pp. 64–99. Available from: http://www.papers2://publication/uuid/51EED98C-39D3-4ECA-9CC8-F7E445CCB145

[43] Jones RM, Somerville LH, Li J, Ruberry EJ, Powers A, Mehta N, et al. Adolescent-specific patterns of behavior and neural activity during social reinforcement learning. Cogn Affect Behav Neurosci. 2014;14(2):683–97. 24550063 10.3758/s13415-014-0257-z PMC4127887

[44] Gläscher J, Hampton AN, O’Doherty JP. Determining a role for ventromedial prefrontal cortex in encoding action-based value signals during reward-related decision making. Cereb Cortex. 2009;19(2):483–95. 18550593 10.1093/cercor/bhn098 PMC2626172

[45] Brock J. Alternative bayesian accounts of autistic perception: comment on Pellicano and Burr. Trends Cogn Sci. 2012;16(12):573–4. 23123383 10.1016/j.tics.2012.10.005

[46] Feldman JI, Dunham K, Cassidy M, Wallace MT, Liu Y, Woynaroski TG. Audiovisual multisensory integration in individuals with autism spectrum disorder: a systematic review and meta-analysis. Neurosci Biobehav Rev. 2018;95:220–34. 30287245 10.1016/j.neubiorev.2018.09.020 PMC6291229

[47] Harms MB, Martin A, Wallace GL. Facial emotion recognition in autism spectrum disorders: a review of behavioral and neuroimaging studies. Neuropsychol Rev. 2010;20(3):290–322. 20809200 10.1007/s11065-010-9138-6

[48] Leung FYN, Sin J, Dawson C, Ong JH, Zhao C, Veic A, et al. Emotion recognition across visual and auditory modalities in autism spectrum disorder: a systematic review and meta-analysis. Dev Rev. 2022;63:1–47.

[49] Todorova GK, Hatton REM, Pollick FE. Biological motion perception in autism spectrum disorder: a meta-analysis. Mol Autism. 2019;10(1):49. 31890147 10.1186/s13229-019-0299-8 PMC6921539

[50] Uljarevic M, Hamilton A. Recognition of emotions in autism: a formal meta-analysis. J Autism Dev Disord. 2013;43(7):1517–26. 23114566 10.1007/s10803-012-1695-5

[51] Peer E, Rothschild D, Gordon A, Evernden Z, Damer E. Data quality of platforms and panels for online behavioral research. Behav Res Methods. 2022;54(4):1643–62. 34590289 10.3758/s13428-021-01694-3 PMC8480459

[52] Peer E, Brandimarte L, Samat S, Acquisti A. Beyond the Turk: alternative platforms for crowdsourcing behavioral research. J Exp Soc Psychol. 2017;70:153–63.

