# Supplementary material for: Do autistic individuals show atypical performance in probabilistic learning? A comparison of cue-number, predictive strength, and prediction error
Source: Mol Autism. 2025 Mar 4;16:15. doi: 10.1186/s13229-025-00651-7 (PMC11877734; doi:10.1186/s13229-025-00651-7)
Supplement: Supplementary file 1 — Supplementary Material 1 [file 13229_2025_651_MOESM1_ESM.docx]

Do autistic individuals show atypical performance in probabilistic learning? A comparison of cue-number, predictive strength, and prediction error.

**ADDITIONAL FILE 1**

Jia Hoong Ong^1^, Lei Zhang^2,3,4^, and Fang Liu^5*^

^1^Department of Psychology, School of Social Sciences, Nottingham Trent University, Nottingham, United Kingdom

^2^Centre for Human Brain Health, School of Psychology, University of Birmingham, Birmingham, United Kingdom

^3^Institute for Mental Health, School of Psychology, University of Birmingham, Birmingham, United Kingdom

^4^Centre for Developmental Science, School of Psychology, University of Birmingham, Birmingham, United Kingdom

^5^School of Psychology and Clinical Language Sciences, University of Reading, Reading, United Kingdom

# Re-analysis of the single-cue and multi-cue tasks using GAMM

Here, we reanalysed the single-cue task and multi-cue task using Generalised Additive Mixed Models, instead of Generalised Linear Mixed Effects models, to explore whether the learning trajectories differed by group and by condition in both tasks.

The approach taken to analyse both tasks are similar. First, trials were divided into four blocks, and the mean score for each block per condition was obtained for each participant. The outcome variable for the model was the mean score. The model included the parametric interaction between group and condition, which was ordered (for single-cue, the reference level was autistic group-deterministic condition; for multi-cue, the reference level was autistic group-ambiguous condition). The model also included a reference smooth for block and a difference smooth for block by group and condition. Random smooths were also incorporated for participants over block. The full model is compared to a null model, where only the reference smooth of block and random participant smooths were included. Both models were fitted using the maximum likelihood method to enable model comparison. Model fitting was done using the *bam()* function of the *mgcv* package. In both tasks, the full model was preferred so, for brevity, the results of the final model are reportedly separately below.

## Single-cue probabilistic learning task

Model output is shown in Table A1.1. The parametric effects revealed that relative to the reference level (i.e., autistic group’s performance on the Deterministic condition), there was no significant differences with the non-autistic group’s performance on the same condition. The autistic group’s performance on the Deterministic condition was significantly better than that of the probabilistic condition for both groups.

**Table A1.1**

*Model output for the single-cue task.*

| **Parametric coefficients** | **Estimate** | **Std. Error** | ***t*** | ***p*** |
| --- | --- | --- | --- | --- |
| (Intercept) | 0.68 | 0.02 | 30.66 | <.001 |
| NT.Deterministic | -0.01 | 0.03 | -0.46 | .644 |
| AS.Probabilistic | -0.08 | 0.02 | -5.54 | <.001 |
| NT.Probabilistic | -0.09 | 0.03 | -2.73 | .007 |
| **Smooth terms** | **edf** | **Ref. df** | ***F*** | ***p*** |
| s(Block) | 2.20 | 2.57 | 12.41 | <.001 |
| s(Block):NT.Deterministic | 1.00 | 1.00 | 0.03 | .861 |
| s(Block):AS.Probabilistic | 1.00 | 1.00 | 5.60 | .018 |
| s(Block):NT.Probabilistic | 1.00 | 1.00 | 5.02 | .025 |
| s(Block, participant) | 88.23 | 412.00 | 1.60 | <.001 |

The smooth terms revealed that the trajectory for the deterministic conditions was not significantly different between the groups (see Figure A1.1). The trajectory for the deterministic condition for the autistic group, however, was significantly different than the probabilistic condition in both groups.


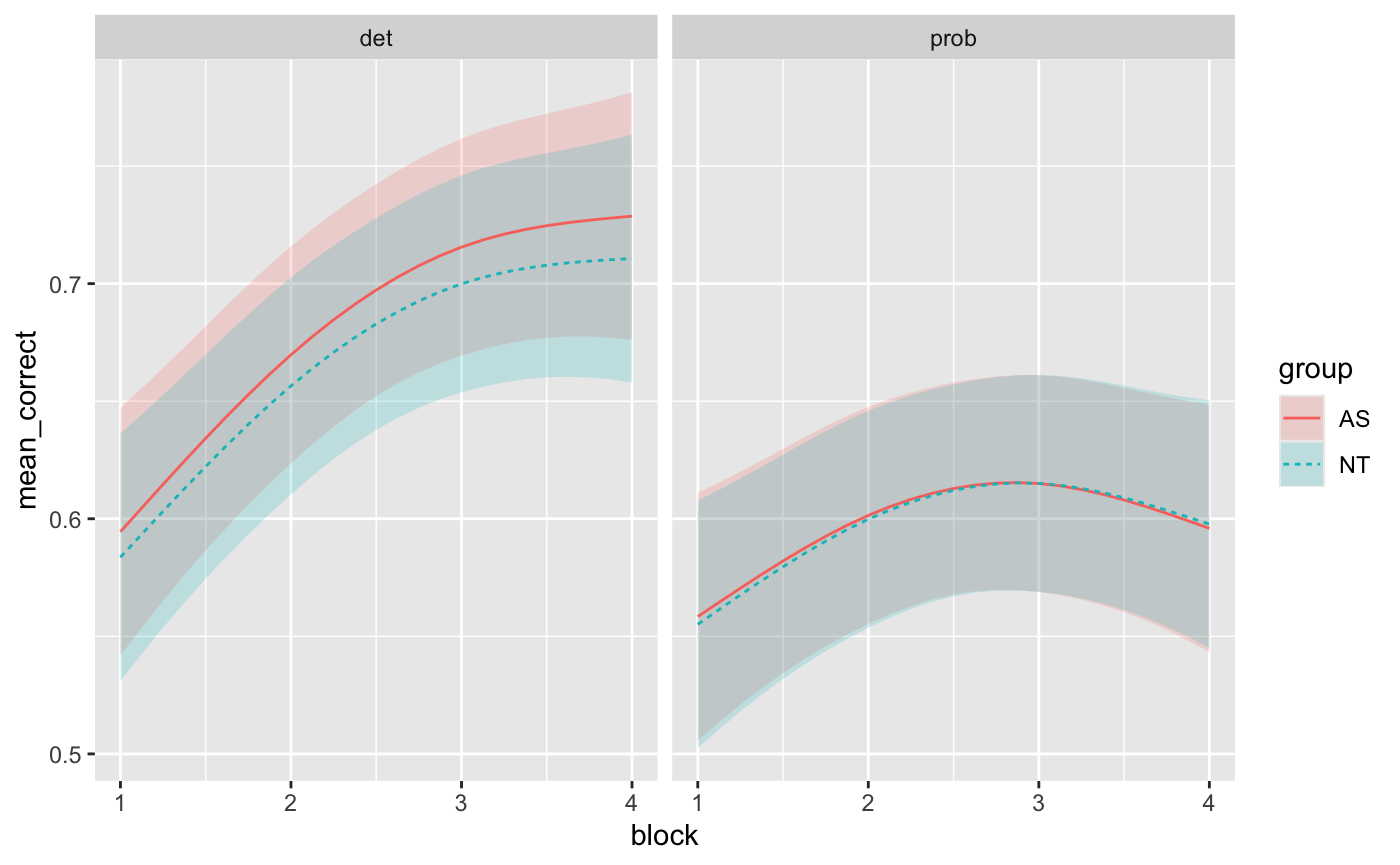


*Figure A1.1*. Smooths estimates from the model as a function of block, condition (deterministic (Det) vs. probabilistic (Prob)), and group (autistic (AS) vs. non-autistic (NT)).

Overall, similar to the findings of the generalised linear mixed effects model reported in the manuscript, there does not seem to be any significant group differences in the single-cue task.

## Multi-cue probabilistic learning task

Table A1.2 displays the model output for the multi-cue task. The parametric effects revealed that relative to the reference level (i.e., autistic group’s performance on the Ambiguous condition), there was no significant differences with the non-autistic group’s overall performance on the same condition. The autistic group’s performance on the Ambiguous condition was significantly poorer than that of the Unambiguous condition for both groups.

**Table A1.2**

*Model output for the multi-cue task.*

| **Parametric coefficients** | **Estimate** | **Std. Error** | ***t*** | ***p*** |
| --- | --- | --- | --- | --- |
| (Intercept) | 0.56 | 0.01 | 41.72 | <.001 |
| NT.Ambiguous | 0.02 | 0.02 | 0.79 | .431 |
| AS.Unambiguous | 0.06 | 0.01 | 5.12 | <.001 |
| NT.Unambiguous | 0.08 | 0.02 | 3.99 | <.001 |
| **Smooth terms** | **edf** | **Ref. df** | ***F*** | ***p*** |
| s(Block) | 1.00 | 1.00 | 2.87 | .091 |
| s(Block):NT.Ambiguous | 1.00 | 1.00 | 5.47 | .020 |
| s(Block):AS.Unambiguous | 1.00 | 1.00 | 8.32 | .004 |
| s(Block):NT.Unambiguous | 1.00 | 1.00 | 2.15 | .143 |
| s(Block, participant) | 119.36 | 412.00 | 1.05 | <.001 |

The smooth terms revealed that the trajectory for the ambiguous condition among the autistic group was significantly different from the non-autistic group (see Figure A1.2). The trajectory for the ambiguous condition and the unambiguous condition for the autistic group was also significantly different. There was no significant difference in the trajectory between the ambiguous condition among the autistic group and the unambiguous condition among the non-autistic group.


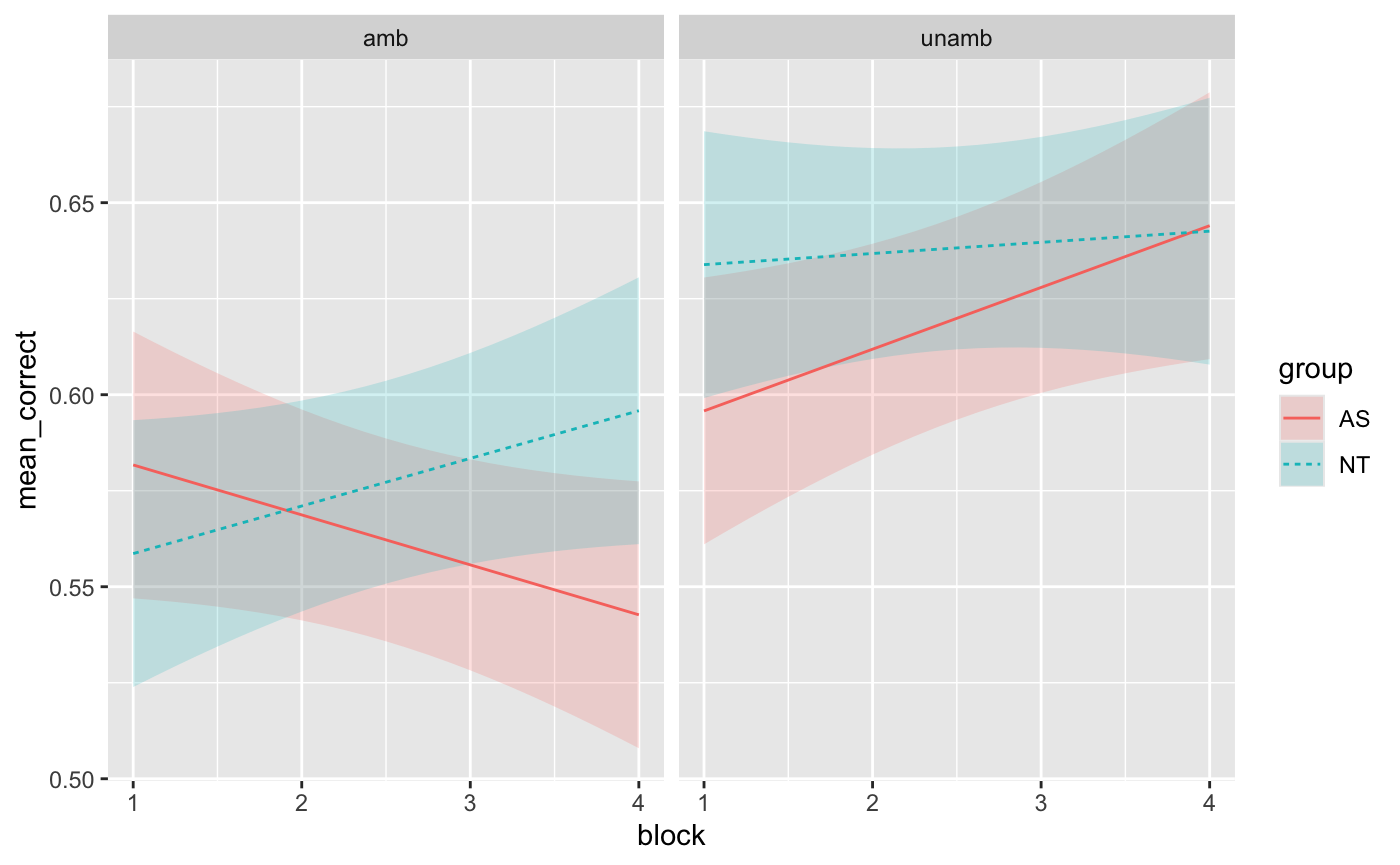


*Figure A1.2*. Smooths estimates from the model as a function of block, condition (ambiguous (Amb) vs. Unambiguous (unamb)), and group (autistic (AS) vs. non-autistic (NT)).

Similar to the results reported in the manuscript, it appears that, for the difficult/ambiguous condition, the overall performance among the non-autistic group was not significantly different from the autistic group. However, their trajectories *were* significantly different; as visualised in Figure A1.2: whereas the non-autistic group appears to be improving over time, the autistic group appears to be performing worse over time. From the figure, it is also clear that towards the end of the task (i.e., Block 4), performance among both groups diverged, though not quite completely, as the 95% confidence intervals still overlap.
